# A New and Improved Sliding Mode Control Design Based on a Grey Linear Regression Model and Its Application in Pure Sine Wave Inverters for Photovoltaic Energy Conversion Systems

**DOI:** 10.3390/mi16040377

**Published:** 2025-03-26

**Authors:** En-Chih Chang, Yeong-Jeu Sun, Chun-An Cheng

**Affiliations:** Department of Electrical Engineering, I-Shou University, No.1, Sec. 1, Syuecheng Rd., Dashu District, Kaohsiung City 84001, Taiwan; enchihchang@isu.edu.tw (E.-C.C.); yjsun@isu.edu.tw (Y.-J.S.)

**Keywords:** new and improved sliding mode control (NISMC), grey linear regression model (GLRM), pure sine wave inverter, photovoltaic (PV) energy conversion system

## Abstract

A new and improved sliding mode control (NISMC) with a grey linear regression model (GLRM) facilitates the development of high-quality pure sine wave inverters in photovoltaic (PV) energy conversion systems. SMCs are resistant to variations in internal parameters and external load disturbances, resulting in their popularity in PV power generation. However, SMCs experience a slow convergence time for system states, and they may cause chattering. These limitations can result in subpar transient and steady-state performance of the PV system. Furthermore, partial shading frequently yields a multi-peaked power-voltage curve for solar panels that diminishes power generation. A traditional maximum power point tracking (MPPT) algorithm in such a case misclassifies and fail to locate the global extremes. This paper suggests a GLRM-based NISMC for performing MPPT and generating a high-quality sine wave to overcome the above issues. The NISMC ensures a faster finite system state convergence along with reduced chattering and steady-state errors. The GLRM represents an enhancement of the standard grey model, enabling greater accuracy in predicting global state points. Simulations and experiments validate that the proposed strategy gives better tracking performance of the inverter output voltage during both steady state and transient tests. Under abrupt load changing, the proposed inverter voltage sag is constrained to 10% to 90% of the nominal value and the voltage swell is limited within 10% of the nominal value, complying with the IEEE (Institute of Electrical and Electronics Engineers) 1159-2019 standard. Under rectified loading, the proposed inverter satisfies the IEEE 519-2014 standard to limit the voltage total harmonic distortion (THD) to below 8%.

## 1. Introduction

In light of recent scientific breakthroughs, photovoltaic (PV) energy conversion systems have emerged as a highly cost-effective long-term investment [1,2]. Maximizing the performance of solar cells is consistently a development focus worldwide, representing a pivotal challenge in PV power generation technology [3]. The achievement of maximum power point tracking (MPPT) in PV systems involves the adjustment of the output from solar cells by means of a direct current (DC) to a DC converter with a MPPT algorithm function. This enables solar panels to output their maximum capacity and achieve fast and accurate tracking [4,5,6]. Then a pure sine wave inverter converts DC to alternating current (AC) when connecting to the grid or supplying AC loads. It necessitates the use of a high-performance pure sine wave inverter based on MPPT to achieve lower AC voltage total harmonic distortion (THD) and quicker dynamic response. Numerous MPPT techniques have been proposed, including perturbation and observation, incremental conductance, and iterative scanning methods [7,8,9]. Particularly, sunlight illumination and ambient climate strongly correlate with the variability of the maximum output power. Traditional MPPT algorithms cannot effectively analyze convergence or track the MPP quickly, resulting in lower output power. Sliding mode control (SMC) resists parametric varying and exogenous perturbations, consequently creating insensitivity. A great number of SMCs are already used in PV energy conversion systems [10,11,12]. A PV system using SMC is subjected to uncertainties with unbounded system state convergence time. This generates chattering and steady state errors, affecting system robustness. Several solutions can mitigate the chattering/steady-state errors, such as perturbation observers and adaptive schemes. These approaches involve relatively sophisticated modeling and time-consuming numerical calculations [13,14]. The quantitative study of one case depicts that both a perturbation observer and adaptive scheme have relatively significant overshoots [15]. However, the performance of a perturbation observer compares favorably with the adaptive scheme.

A new and improved sliding mode control (NISMC) provides a bounded system state convergence time, less chattering, and reduced steady-state error rejection [16,17]. Its sliding surface improves conventional SMC weakness and strengthens system control behavior [18,19,20]. Although NISMC does give the desired control response, the PV arrays tend to be partially shaded due to environmental concerns. This can greatly suppress the PV system’s power output, causing it to incur massive energy losses. Namely, the output power of the PV array oscillates erratically with multiple localized extremes. Using traditional MPPT algorithms, it only finds local maximum power points instead of global ones. A few methods are available for handling the multiple local extremum issue, such as Tabu search and greedy algorithms [21,22]. The Tabu search method can yield optimal solutions, with the exception of often taking a lengthy executing time. The greedy algorithm has a greater speed of convergence, but it restricts itself to a localized search. A case report on quantitative analysis has indicated that the Tabu search method or the greedy algorithm alone cannot achieve the optimal response [23]. However, if both of them are combined, it may be a suitable solution for the case. The grey prediction (GP) technique was created in the 1980s, and has been successfully implemented in various technical domains. It has efficiently tackled the prediction challenges of uncertain systems [24,25]. The GP uses historical and current data to illustrate and analyze the future trend of series numbers for dynamic systems. A grey model (GM) can be built using the system output signal and a minor amount of sample data. The GM generally operates in cases where the series data display a regular trend of the exponential function. It is difficult to predict the final result when there are irregular changes in the series data or the trend of complex functions. In the application of converters and inverters, the predicted trend of the traditional GM sequence loses its precision due to sudden load changing and nonlinear load effects. The grey linear regression model (GLRM) has been widely used in many trend forecasting analyses. A GLRM using the combined effect with exponential and linear functions can enhance the accuracy of prediction [26,27,28]. The combination of NISMC and GLRM for pure sine wave inverters is a good suggestion. The GLRM is derived from the five latest voltage values to predict the state of the next voltage. The NISMC will then generate a control force to quickly converge the tracking error (the difference between the predicted state value and the reference voltage) towards the origin. The remarkable contributions of the closed-loop control technology presented in this paper can be highlighted in the following: (i) mitigation of chattering; (ii) reduction of steady-state error; (iii) shortening convergence time; and (iv) establishing an as low as possible THD. Simulations and experiments reveal that the proposed inverter leads to the improvement of steady-state and transient performance when exposed to variety of loading conditions. The paper is structured as follows: Section 1 surveys the constraints related to classical sliding mode control and conventional gray prediction. Then, the recommendations designed to improve inverter performance are presented. The dynamic models of the converter and inverter are analyzed in Section 2. The design of the proposed control technology is described in Section 3. Simulations and experiment results are given in Section 4 for validating the effect of the proposed control technology. Section 5 provides a synthesis of the discussion and analysis. Section 6 summarizes the concluding remarks of this paper.

## 2. System Description

**Figure 1:**
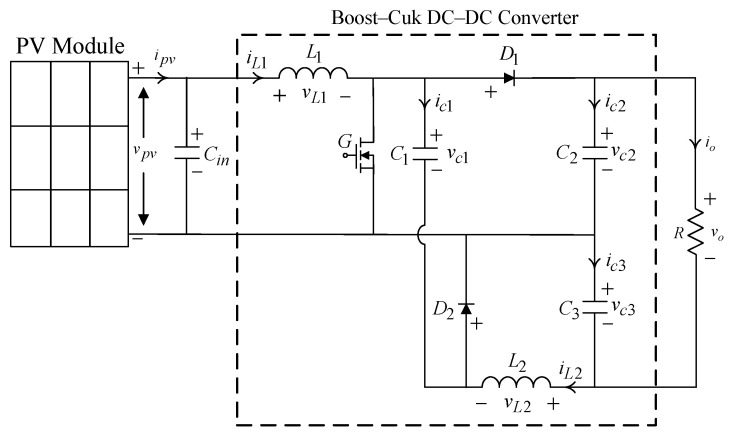
Circuit of the Boost–Cuk DC–DC converter.

A PV energy conversion system comprises several fundamental components that collaboratively facilitate the conversion of solar energy into electrical energy. Initially, the system includes a solar panel, which is tasked with capturing solar radiation. Subsequently, a DC–DC converter is employed to regulate the voltage to an appropriate level. Following this, a pure sine wave DC–AC inverter is utilized to convert the DC into AC, enabling connection to the electrical load. The system is engineered to continuously optimize its performance in response to variations in sunlight and temperature. It can control the voltage as well, ensuring that it stays within the specified range. To achieve this, a Boost–Cuk DC–DC converter is required, as illustrated in Figure 1. This high step-up converter consists of a hybrid configuration of a Boost converter and a Cuk converter. Its advantages include higher voltage gain, less voltage stress, continuous input and output currents, and non-inverting output [29,30]. The Boost–Cuk DC–DC converter is therefore crucial in adjusting the voltage output of the solar panel, maximizing energy efficiency.

**Figure 2:**
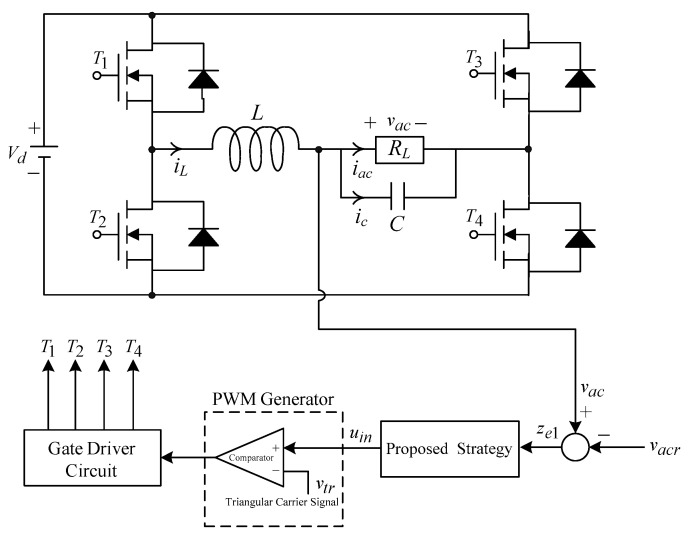
Structure of a pure sine wave inverter.

The variables $i_{pv}$ and $v_{pv}$ are the current and voltage of the PV module, respectively. The variable $i_{L1}$ represents the current flowing through the step-up inductor $L_{1}$ on the input side, and the variable $v_{L1}$ is the voltage across the inductor. The inductor $L_{2}$ has a current $i_{L2}$ that flows through it. The variable $v_{L2}$ stands for the voltage across the inductor. The capacitor $C_{1}$ has a current $i_{c1}$ and a voltage $v_{c1}$ across the capacitor. There are two capacitors, $C_{2}$ and $C_{3}$, at the output across the load R. The currents, $i_{c2}$, $i_{c3}$, and $i_{o}$, flow through two capacitors and the load whose cross-voltages are $v_{c2}$, $v_{c3}$, and $v_{o}$, respectively. Then, the dynamic equation for the Boost–Cuk DC–DC converter is constructed using the state–space averaging technique, which is described below:

(1) $$\dot{x}=A_{bk}x+B_{bk}u_{bk}$$

where $\dot{x}={\left[\begin{array}{ccccc} {\dot{i}}_{L1} & {\dot{i}}_{L2} & {\dot{v}}_{c1} & {\dot{v}}_{c2} & {\dot{v}}_{c3} \end{array}\right]}^{T}$, $A_{bk}=A_{bk1}D+A_{bk2}(1-D)$, $B_{bk}=B_{bk1}D+B_{bk2}(1-D)$, $A_{bk1}=\left[\begin{array}{ccccc} 0 & 0 & 0 & 0 & 0\\ 0 & 0 & -\frac{1}{L_{2}} & 0 & 1\\ 0 & \frac{1}{C_{1}} & 0 & 0 & 0\\ 0 & 0 & 0 & -\frac{1}{RC_{2}} & -\frac{1}{RC_{2}}\\ 0 & -\frac{1}{RC_{3}} & 0 & -\frac{1}{RC_{3}} & -\frac{1}{RC_{3}} \end{array}\right]$, $A_{bk2}=\left[\begin{array}{ccccc} 0 & 0 & 0 & -\frac{1}{L_{1}} & 0\\ 0 & 0 & 0 & 0 & 1\\ \frac{1}{C_{1}+C_{2}} & 0 & -\frac{1}{R(C_{1}+C_{2})} & -\frac{1}{R(C_{1}+C_{2})} & 0\\ \frac{1}{C_{1}+C_{2}} & 0 & -\frac{1}{R(C_{1}+C_{2})} & -\frac{1}{R(C_{1}+C_{2})} & 0\\ 0 & -\frac{1}{C_{3}} & 0 & -\frac{1}{RC_{3}} & -\frac{1}{RC_{3}} \end{array}\right]$, $B_{bk1}={\left[\begin{array}{ccccc} \frac{V_{pv}}{L_{1}} & 0 & 0 & 0 & 0 \end{array}\right]}^{T}$, $B_{bk2}={\left[\begin{array}{ccccc} \frac{V_{pv}}{L_{1}} & 0 & 0 & 0 & 0 \end{array}\right]}^{T}$, D denotes duty cycle, and $u_{bk}$ represents the control input. The variable $v_{dcr}$ is defined as the voltage reference value corresponding to the maximum power point established by the GLRM algorithm. The NISMC closed-loop control strategy is crucial to ensure the alignment of variables $v_{pv}$ and $v_{dcr}$. The output voltage error is characterized as the state variable $e_{1}=v_{pv}-v_{dcr}$. The stability of Equation (1) will be guaranteed by a well-crafted control law, enabling the error $e_{1}$ to quickly approach the equilibrium point. Consequently, the output voltage from the PV system will align with the required reference value. The tracking control system can maintain its speed and accuracy, even in the case of partial shading from the PV MPPT system. Figure 2 illustrates a pure sine wave inverter, which comprises four semiconductor switches, inductor–capacitor (LC) filters, and a load, $R_{L}$. The conversion of the generated DC power into AC power for load supply is achieved via a pure sine wave DC–AC inverter. It has the advantages of simple structure and high efficiency, as well as lower cost, compared to three-phase inverters [31,32].

The DC-link voltage is denoted as $V_{d}$. The variables $i_{L}$ and $i_{c}$ represent the currents flowing through the inductor L and capacitor C, respectively. The output voltage and current are indicated by $v_{ac}$ and $i_{ac}$, respectively. Kirchhoff’s laws of voltage and current are applied to Figure 2, as well as letting state variables $z_{1}=v_{ac}$ and $z_{2}={\dot{v}}_{ac}$. The dynamic behavior of the pure sine wave inverter equipped with an output LC filter can be articulated as follows:

(2) $$\left\{\begin{array}{l} {\dot{z}}_{1}=z_{2}\\ {\dot{z}}_{2}=-\frac{1}{LC}z_{1}-\frac{1}{R_{L}C}z_{2}+\frac{{\bar{G}}_{pwm}}{LC}u_{in} \end{array}\right.$$

where ${\bar{G}}_{pwm}=V_{d}/{\hat{v}}_{tr}$ signifies the scaled constant gain of the inverter, ${\hat{v}}_{tr}$ denotes the amplitude of the triangular carrier signal, and $u_{in}$ represents a control input. The pulse width modulation (PWM) signals controlling the inverter’s switches ($T_{1,}\;T_{2,}\;T_{3,}\;T_{4}$) are generated by comparing $u_{in}$ with a triangular carrier signal $v_{tr}$. The design of a pure sine wave inverter is fundamentally a conventional servo control problem. The output voltage of the inverter will be a sine wave, which is required to track the desired AC referred signal, $v_{acr}$. The error state variables can be described as an expression of the correlation between the (2) and $v_{acr}$:

(3) $$\left\{\begin{array}{c} z_{e1}=z_{1}-v_{acr}\;\\ z_{e2}=z_{2}-{\dot{v}}_{acr}\; \end{array}\right.$$

where $v_{acr}=V_{m}\sin(2\pi ft)$, $V_{m}$ refers to the maximum value, and f corresponds to the frequency.

**Figure 3:**
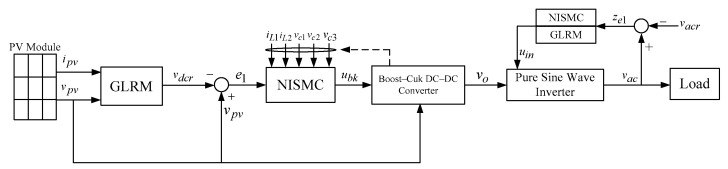
Block diagram of the overall control system.

By utilizing (2) and (3), the error dynamic equation for the pure sine wave inverter can be formulated as follows:

(4) $$\left\{\begin{array}{l} {\dot{z}}_{e1}=z_{e2}\\ {\dot{z}}_{e2}=a_{1}z_{e1}+a_{2}z_{e2}+b_{in}u_{in}+N \end{array}\right.$$

where $a_{1}=-1/LC$, $a_{2}=-1/R_{L}C$, $b_{in}={\bar{G}}_{pwm}/LC$, and $N=-a_{1}v_{acr}-a_{2}{\dot{v}}_{acr}-{\ddot{v}}_{acr}$ are utilized to specify external disturbances produced by step load changes or nonlinear loads. The $u_{in}$ presented in (4) can be designed to exhibit a tracking behavior that approaches the equilibrium point. Specifically, the NISMC is designed to regulate the output voltage of an inverter, thereby minimizing the error trajectory to zero during a short period of time. The block diagram of the overall control system is displayed in Figure 3. The subsequent section presents both the illustration and derivation of the control design.

## 3. Control Design

In relation to the error state, Equation (4), a sliding surface must be created to facilitate rapid limited time convergence with no singularities. This surface can be articulated as follows, where ε is greater than zero and $\kappa_{1}$ and $\kappa_{2}$ have positive odd numbers in the limit $1<\frac{\kappa_{1}}{\kappa_{2}}<2$:

(5) $$\sigma =z_{e1}+\frac{1}{\varepsilon }z_{e2}^{\kappa_{1}/\kappa_{2}}$$

To promote swift entry into the sliding surface, a NISMC is suggested below:

(6) $$\dot{\sigma }=-\rho_{1}{\left(\left|z_{e}\right|\left|\sigma \right|\right)}^{\gamma_{1}}\tanh^{-1}(\sigma /\zeta )-\rho_{2}{\left|\sigma \right|}^{\gamma_{2}}\mathrm{gd}(\sigma /\delta )-\rho_{3}{\left|\sigma \right|}^{\gamma_{3}}\sigma$$

where $\rho_{1}>0$, $\rho_{2}>0$, $\rho_{3}>0$, $0<\gamma_{1}<1$, $1<\gamma_{2}<2$, $\gamma_{3}>0$, ζ>0, δ>0, and $\tanh^{-1}(\cdot )$ and gd(⋅) represent the saturation functions based on the inverse hyperbolic tangent and the Gudermannian function, respectively.

Following Equations (4)–(6), the control law $u_{in}$ for the NISMC can be deduced as follows:

(7) $$u_{in}=-b_{in}^{-1}[a_{1}z_{e1}+a_{2}z_{e2}+\frac{\varepsilon \kappa_{2}}{\kappa_{1}}z_{e2}^{\kappa_{1}/\kappa_{2}}+\rho_{1}{\left(\left|z_{e}\right|\left|\sigma \right|\right)}^{\gamma_{1}}\tanh^{-1}(\sigma /\zeta )+\rho_{2}{\left|\sigma \right|}^{\gamma_{2}}\mathrm{gd}(\sigma /\delta )+\rho_{3}{\left|\sigma \right|}^{\gamma_{3}}\sigma ]$$

**Proof.**  A candidate for the Lyapunov function can be defined as follows:

(8) $$V=0.5\cdot \sigma^{2}$$

By applying the time derivative to trajectory, Equation (8) in conjunction with control law (7), the subsequent equation can be derived as follows:

(9) $$\begin{array}{ll} \dot{V} & =\sigma \dot{\sigma }\\ & =\sigma {\left(z_{e1}+\frac{1}{\varepsilon }z_{e2}^{\kappa_{1}/\kappa_{2}}\right)}^{\prime }\\ & \leq -\rho_{1}{\left(\left|z_{e}\right|\left|\sigma \right|\right)}^{\gamma_{1}}\tanh^{-1}(\sigma /\zeta )-\rho_{2}{\left|\sigma \right|}^{\gamma_{2}}\mathrm{gd}(\sigma /\delta )-\rho_{3}{\left|\sigma \right|}^{\gamma_{3}}\sigma \end{array}$$

☐

It is evident from Equation (9) that the derivative is not equal to zero concerning variables σ and $z_{e}$, leading to the conclusion that $\dot{V}$ is less than zero. In the publications [33,34], the finite time convergence of σ has been verified. Namely, the time $t_{a}=(\kappa_{1}{\left|z_{e1}(0)\right|}^{1-\kappa_{2}/\kappa_{1}})/\left(\varepsilon^{\kappa_{2}/\kappa_{1}}(\kappa_{1}-\kappa_{2})\right)$ for the state to attain the equilibrium point is bounded. The variable $\dot{\sigma }$ enables the state to rapidly approach the sliding surface in a finite amount of time that can be explained below: The state trajectory for reaching the sliding surface is divided into two phases. One term $-\rho_{1}{\left(\left|z_{e}\right|\left|\sigma \right|\right)}^{\gamma_{1}}\tanh^{-1}(\sigma /\zeta )$ denotes the quality of the dynamic behavior exhibited by the state trajectory approaching the sliding surface. The other term $-\rho_{2}{\left|\sigma \right|}^{\gamma_{2}}\mathrm{gd}(\sigma /\delta )$ concerns the quality of the dynamic behavior while the state trajectory departs from the sliding surface. The term $-\rho_{3}{\left|\sigma \right|}^{\gamma_{3}}\sigma$ gives a fine adjustment of the approval rate of the aforementioned two terms. In order to mitigate the exceedingly high magnitude from the input signal, both $\tanh^{-1}(\sigma /\zeta )$ and gd(σ/δ) act as the smoothing function. Additionally, the GLRM algorithm is employed to find the optimal extremes to maintain the system’s maximum energy efficiency. Although the advantages of the proposed strategy are greater than the conventional SMC, the complexity of implementation should be considered. In this paper, a high-speed digital signal processor (DSP) will be used to implement the proposed strategy. It can perform rapid sampling of the output and has a fast computation speed, thus solving the complexity issue of the implementation. The operating procedures for the GLRM can be summarized below:

Step 1: The original data sequence ${Χ}^{\left(0\right)}$ (which represents output voltage values) must be represented as follows:

(10) $${Χ}^{\left(0\right)}=\left\{\chi^{\left(0\right)}(1)\;,\;\chi^{\left(0\right)}(2),\cdots ,\;\chi^{\left(0\right)}(j)\right\}$$

where j denotes the amount of data.

Step 2: One way to express the accumulated generating operation (AGO) can be expressed as follows:

(11) $$\chi^{\left(1\right)}(k)=\sum_{m=1}^{k}\chi^{\left(0\right)}(m)$$

where k=1, 2,⋯, j.

Step 3: The following findings can be obtained by using ${Χ}^{\left(1\right)}$ to create a first-order differential grey model:

(12) $$\frac{d\chi^{\left(1\right)}}{dt}+p\chi^{\left(1\right)}=q$$

where p and q stand for the model parameters.

Using the mean generation operation to ${Χ}^{\left(1\right)}$, the requisite data sequence for obtaining the corresponding grey background value is derived as follows:

(13) $${Ζ}^{\left(1\right)}(k)=\left[\chi^{\left(1\right)}(k-1)+\chi^{\left(1\right)}(k)\right]/2$$

where k=2, 3,⋯, j.

The following expression is obtained by reformulating Equation (12) into a discrete sequence:

(14) $$\chi^{\left(0\right)}(k)+p{Ζ}^{\left(1\right)}(k)=q$$

Finding p and q using the least squares method yields the following:

(15) $$\left[\begin{array}{c} p\\ q \end{array}\right]={\left(B^{T}B\right)}^{-1}B^{T}Y$$

where $B=\left[\begin{array}{cc} -{Ζ}^{\left(1\right)}(2) & 1\\ -{Ζ}^{\left(1\right)}(3) & 1\\ \cdot & \cdot \\ \begin{array}{l} \cdot \\ \cdot \end{array} & \begin{array}{l} \cdot \\ \cdot \end{array}\\ -{Ζ}^{\left(1\right)}(j) & 1 \end{array}\right]$, and $\;Y=\left[\begin{array}{c} \chi^{\left(0\right)}(2)\\ \chi^{\left(0\right)}(3)\\ \cdot \\ \begin{array}{l} \cdot \\ \cdot \end{array}\\ \chi^{\left(0\right)}(j) \end{array}\right]$.

By replacing p and q in Equation (12), the time response can be derived as follows:

(16) $${\hat{\chi }}^{\left(1\right)}(k+1)=K_{1}e^{-\phi k}+K_{2}$$

where $K_{1}=\chi^{\left(0\right)}(1)-\frac{q}{p}$, $K_{2}=\frac{q}{p}$, and ϕ=−p. To sufficiently reflect the system’s varying characteristics and reduce the randomness, a GLRM can be used to construct ${\hat{\chi }}^{\left(1\right)}(k)$. Thus, Equation (16) can be rewritten as follows:

(17) $${\hat{\chi }}^{\left(1\right)}(k+1)=K_{1}e^{-\phi k}+K_{2}k+K_{3}$$

where $K_{1}$, $K_{2}$, $K_{3}$, and ϕ stand for the model parameters that must be established.

Let $Ζ(k)={\hat{\chi }}^{\left(1\right)}(k+1)-{\hat{\chi }}^{\left(1\right)}(k)$ (k=1, 2, ⋯, j−1), and assume $Y_{n}(k)=Z(k+n)-Z(k)$ (n=1, 2, ⋯, j−3 and k=1, 2, ⋯, j−n−2) and $e^{\phi k}=\frac{Y_{n}(k+1)}{Y_{n}(k)}$. Then, it obtains

(18) $$\phi =\ln\frac{Y_{n}(k+1)}{Y_{n}(k)}$$

Replacing ${\hat{\chi }}^{\left(1\right)}$ in Equation (17) with $\chi^{\left(1\right)}$ gives an approximate solution $\tilde{\phi }$ from ϕ. Taking different values of n yields a variety of values of $\tilde{\phi }$ in addition to adopting their average as an estimate of ϕ. Accordingly, $\tilde{\phi }$ can be decided as follows:

(19) $$\tilde{\phi }=\frac{\sum_{n=1}^{n-3}\;\sum_{k=1}^{n-j-2}{\tilde{\phi }}_{n}(k)}{(n-2)(j-3)/2}$$

where ${\tilde{\phi }}_{n}(k)$ represents the value of ϕ at various step sizes n and different moments k. The coefficients $K_{1}$, $K_{2}$, and $K_{3}$ can be obtained by the least squares method. Given that $H(t)=e^{\hat{\phi }k}$, then ${\hat{\chi }}^{\left(1\right)}(k)=K_{1}H(t)+K_{2}k+K_{3}$, and the following result can be derived as follows:

(20) $$K=\left[\begin{array}{c} K_{1}\\ K_{2}\\ K_{3} \end{array}\right]={\left({Μ}^{T}Μ\right)}^{-1}{Μ}^{T}{Χ}^{\left(1\right)}$$

where $Μ=\left[\begin{array}{ccc} H(1) & 1 & 1\\ H(2) & 2 & 1\\ \vdots & \vdots & \vdots \\ H(j) & j & 1 \end{array}\right]$, and $\;{Χ}^{\left(1\right)}=\left[\begin{array}{c} \chi^{\left(0\right)}(2)\\ \chi^{\left(0\right)}(3)\\ \cdot \\ \begin{array}{l} \cdot \\ \cdot \end{array}\\ \chi^{\left(0\right)}(j) \end{array}\right]$.

The forecast output can be obtained as follows:

(21) $${\hat{\chi }}^{\left(1\right)}(k)=e^{\hat{\phi }k}+K_{2}k+K_{3}$$

Step 4: The inverse accumulated generating operation (IAGO) can be used to calculate the expected output at (k+1):

(22) $${\hat{\chi }}^{\left(0\right)}(k+1)={\hat{\chi }}^{\left(1\right)}(k+1)-{\hat{\chi }}^{\left(1\right)}(k)$$

This means that if $K_{1}=0$, the AGO series is a GLRM, whereas when $K_{2}=0$, the AGO series becomes a traditional grey model. That is to say, such a modified model reflects the characteristics of linear factors and depicts the index growing tendency of nonlinear changes, thereby improving the forecasting accuracy.

## 4. Simulation and Experimental Results

**Figure 4:**
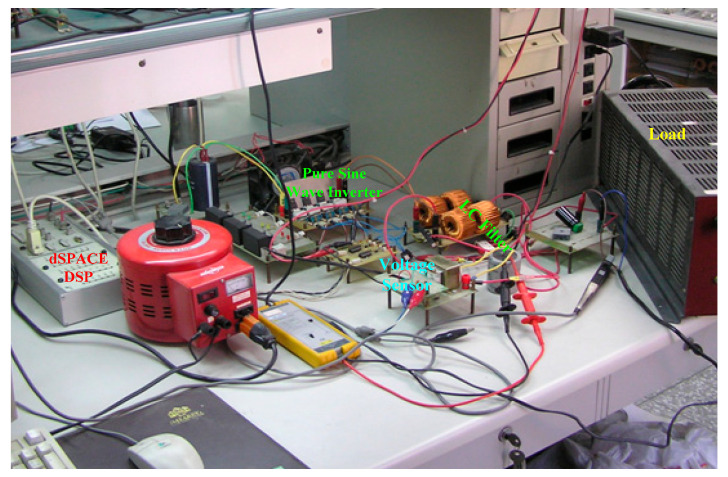
Photograph of the experimental hardware setup.

**Figure 5:**
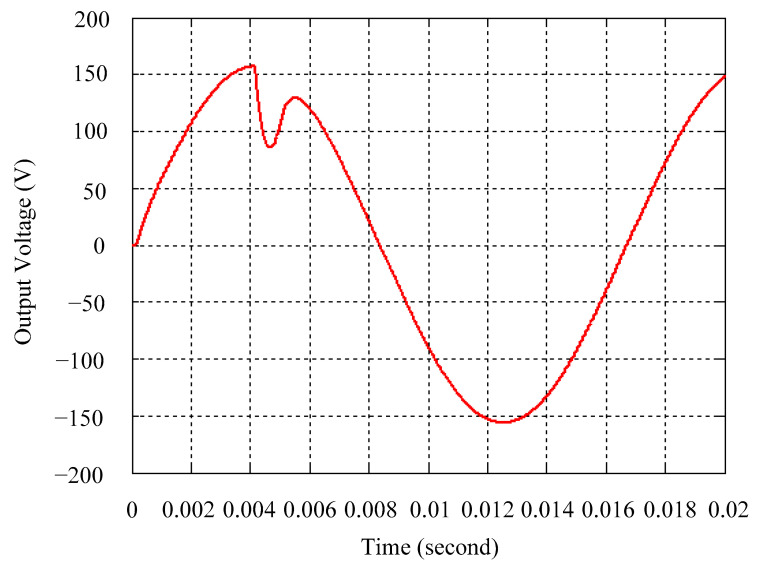
Simulated sine wave output voltage of the conventional SMC at abrupt load increasing.

**Figure 6:**
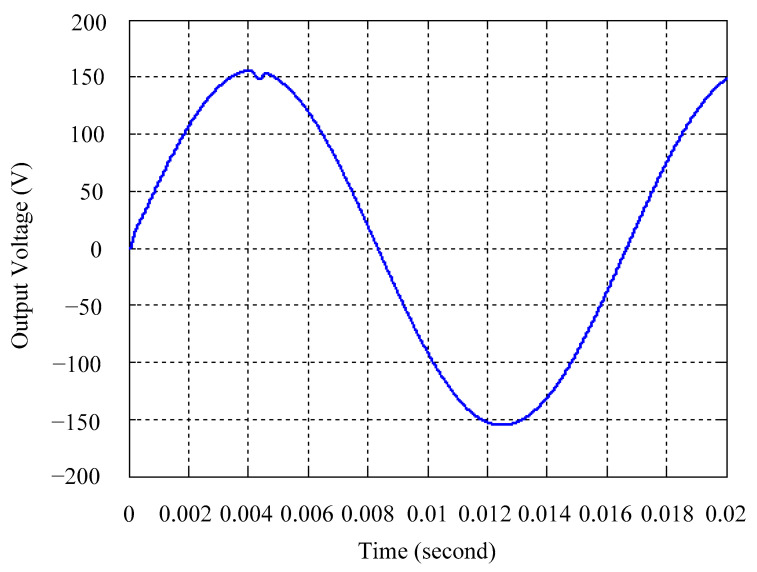
Simulated sine wave output voltage of the proposed strategy at abrupt load increasing.

**Figure 7:**
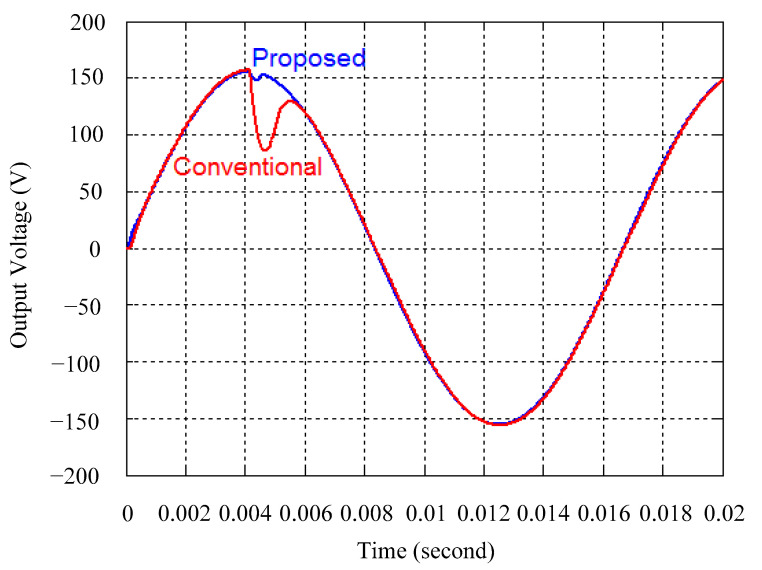
Comparison of the difference in the simulated sine wave output voltage between the conventional SMC and the proposed strategy at abrupt load increasing.

**Figure 8:**
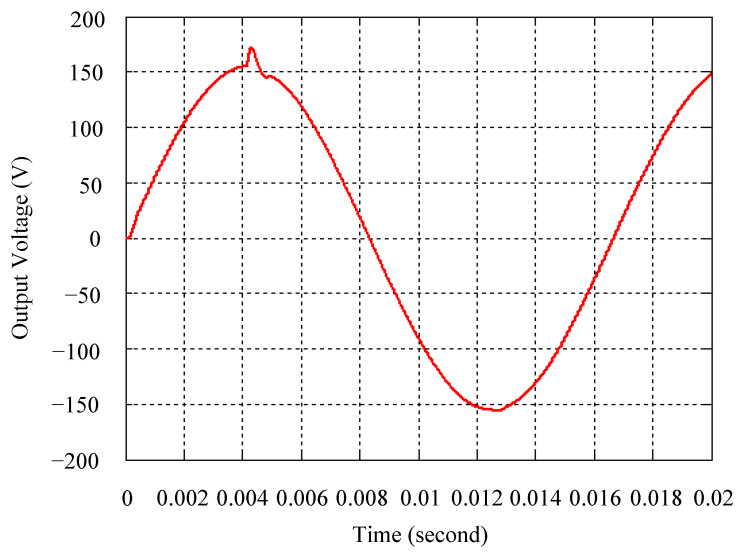
Simulated sine wave output voltage of the conventional SMC at abrupt load removing.

**Figure 9:**
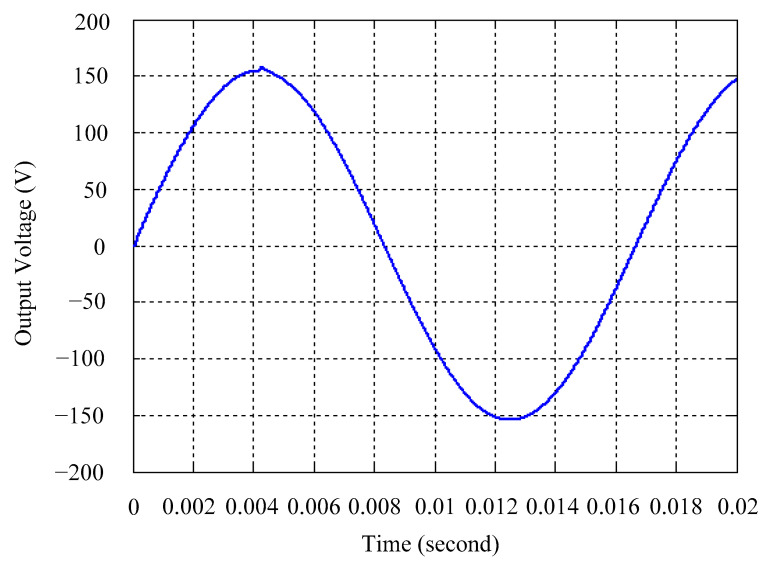
Simulated sine wave output voltage of the proposed strategy at abrupt load removing.

**Figure 10:**
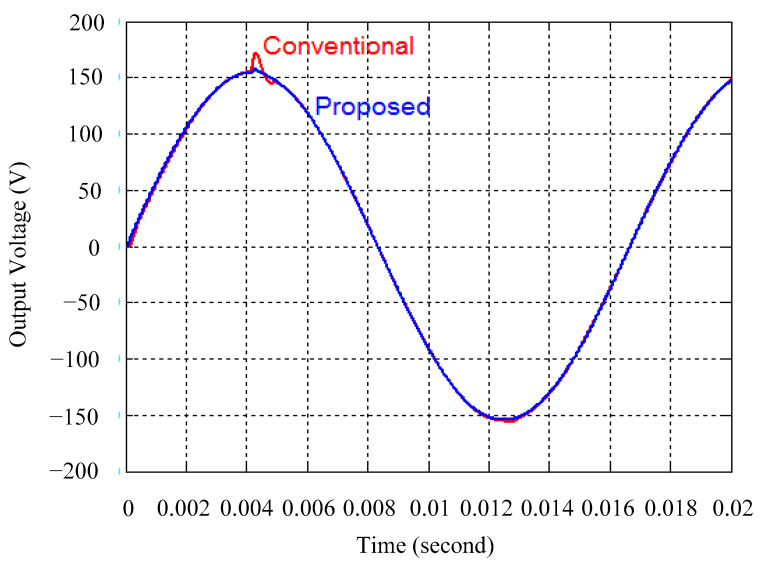
Comparison of the difference in the simulated sine wave output voltage between the conventional SMC and the proposed strategy at abrupt load removing.

**Figure 11:**
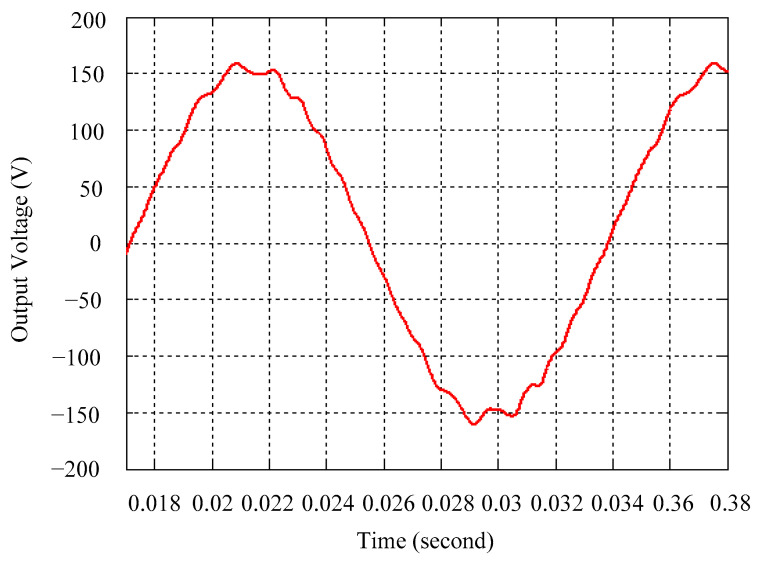
Simulated sine wave output voltage of the conventional SMC at rectified loading.

**Figure 12:**
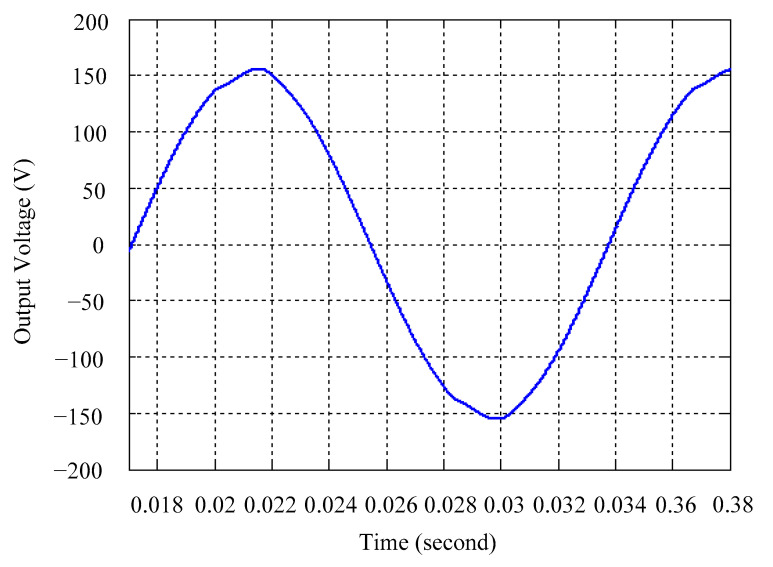
Simulated sine wave output voltage of the proposed strategy at rectified loading.

**Figure 13:**
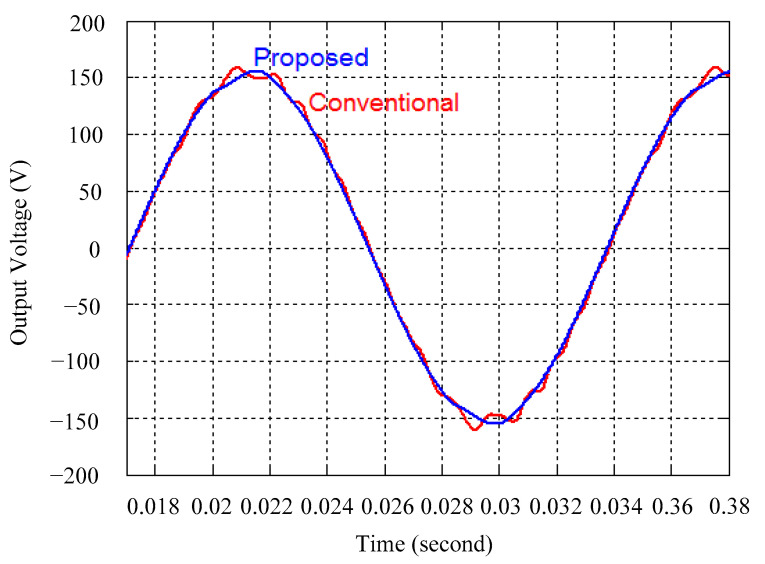
Comparison of the difference in the simulated sine wave output voltage between the conventional SMC and the proposed strategy at rectified loading.

**Figure 14:**
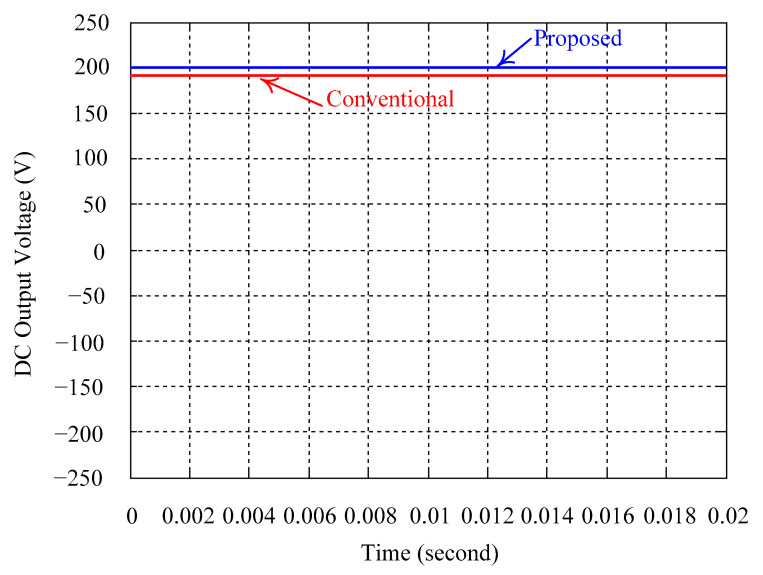
Comparison of the simulated DC output voltage from the converter between the conventional SMC and the proposed strategy under partial shading.

**Figure 15:**
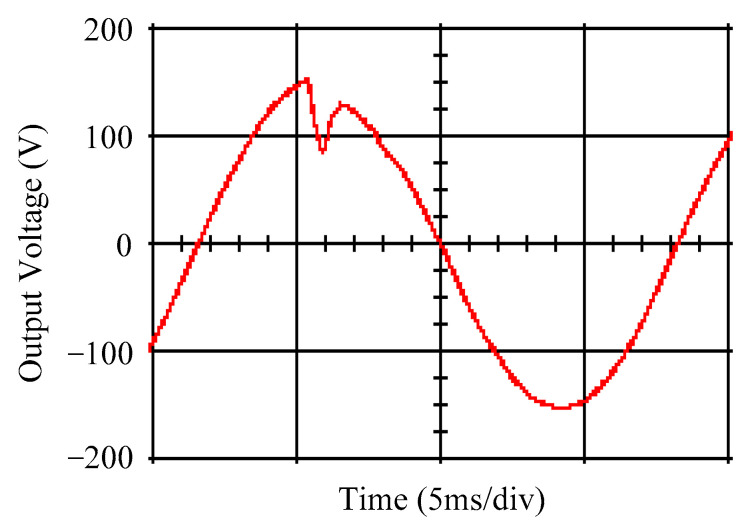
Experimental sine wave output voltage of the conventional SMC at abrupt load increasing.

**Figure 16:**
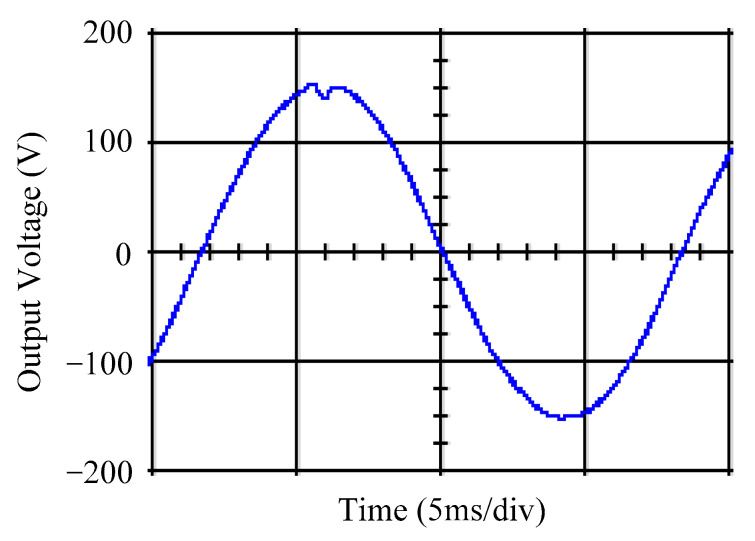
Experimental sine wave output voltage of the proposed strategy at abrupt load increasing.

**Figure 17:**
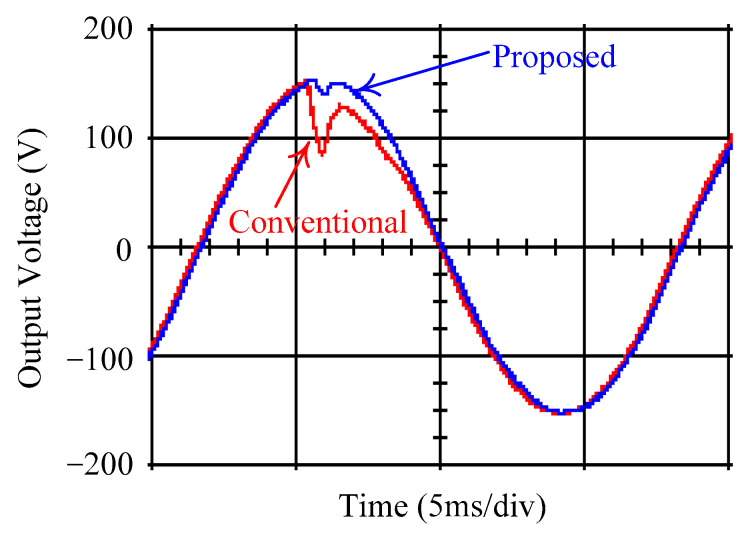
Comparison of the difference in experimental sine wave output voltage between the conventional SMC and the proposed strategy at abrupt load increasing.

**Figure 18:**
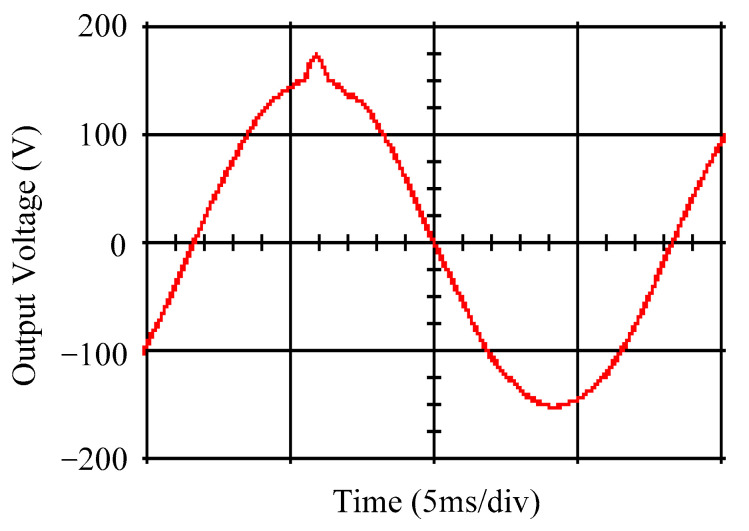
Experimental sine wave output voltage of the conventional SMC at abrupt load removing.

**Figure 19:**
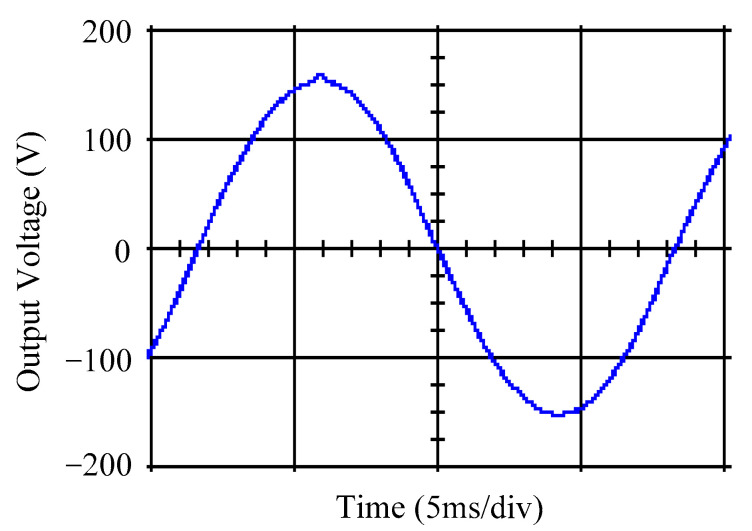
Experimental sine wave output voltage of the proposed strategy at abrupt load removing.

**Figure 20:**
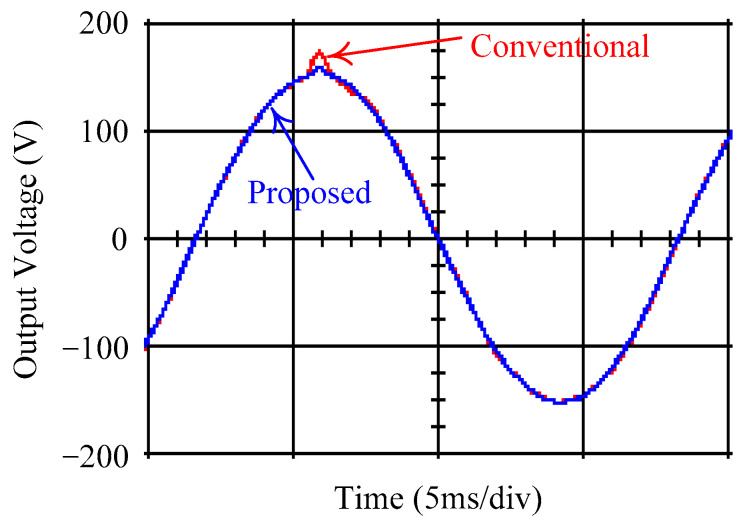
Comparison of the difference in experimental sine wave output voltage between the conventional SMC and the proposed strategy at abrupt load removing.

**Figure 21:**
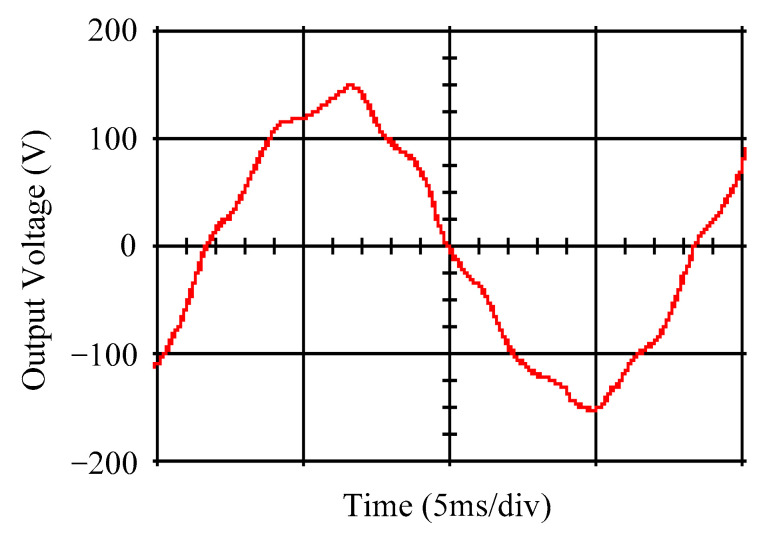
Experimental sine wave output voltage of the conventional SMC at rectified loading.

**Figure 22:**
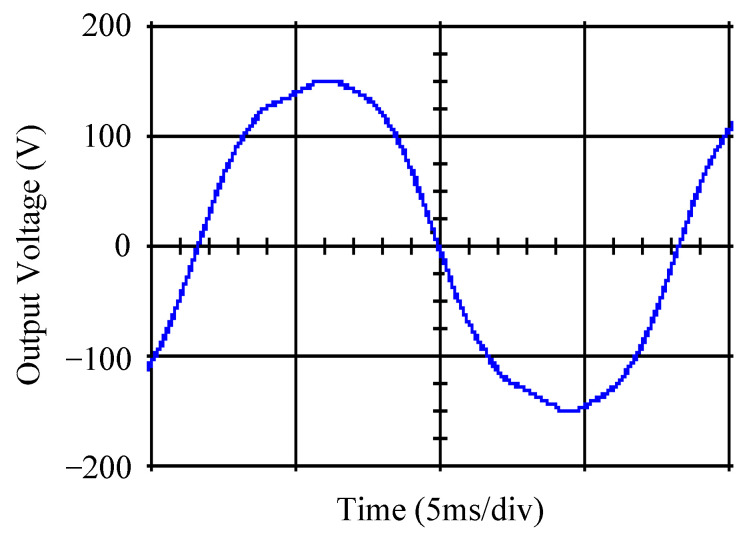
Experimental sine wave output voltage of the proposed strategy at rectified loading.

**Figure 23:**
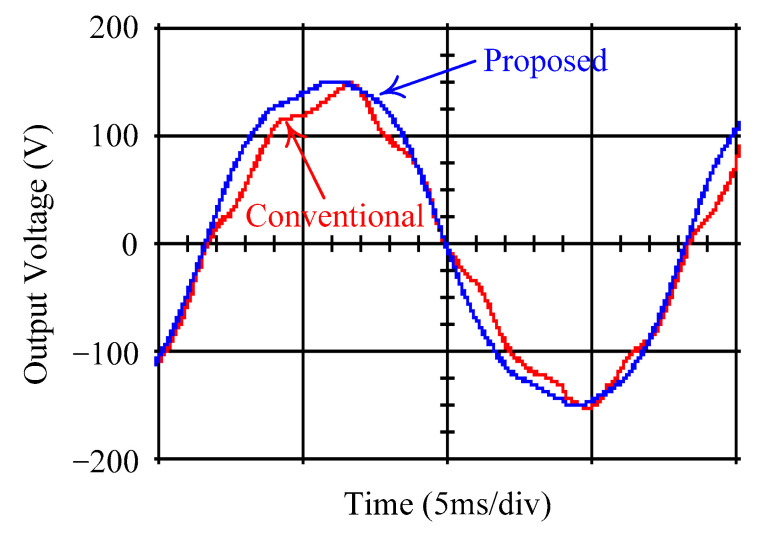
Comparison of the difference in experimental sine wave output voltage between the conventional SMC and the proposed strategy at rectified loading.

**Figure 24:**
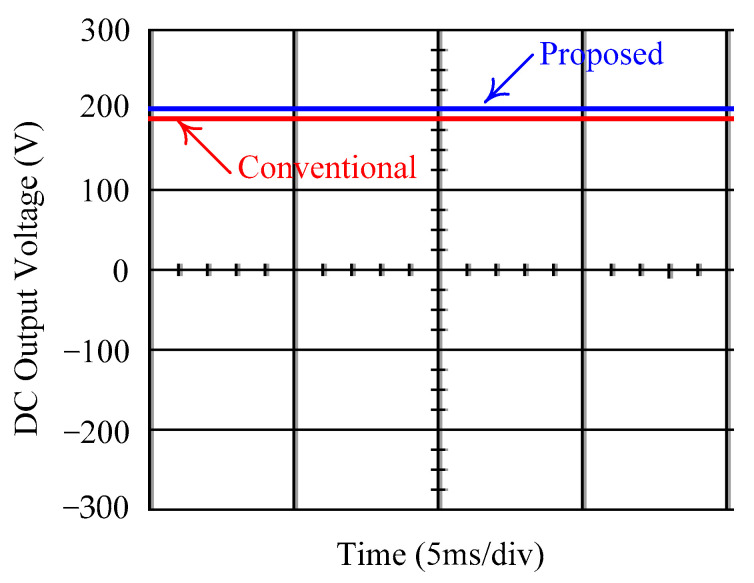
Comparison of experimental DC output voltage from the converter between the conventional SMC and the proposed strategy under partial shading.

As delineated in Table 1, the system parameters are enumerated in detail. Both the proposed algorithm and the PWM modules are developed in Matlab (version 6.1)/Simulink (version 4.1) software. The pure sine wave inverter is implemented on a dSPACE (dSPACE GmbH, Paderborn, Germany) with high-speed DSP. The proposed algorithm can be directly converted into C codes from Simulink diagrams, allowing the implementation on the dSPACE hardware. In order to isolate the control and power circuits, four optocouplers (PC923) are required. The power MOSFET selected is the IRF460, and the AD202 isolation amplifier is adopted as the voltage sensor. Figure 4 depicts the photograph of the experimental hardware setup. Figure 5 illustrates the simulated sine wave output voltage produced by the conventional SMC method from no load to full load. The waveform demonstrates a notable voltage decline, followed by longer recovery duration. Figure 6 presents the simulated sine wave output voltage produced by the proposed strategy from no load to full load. The proposed strategy exhibits a quick and instantaneous response time and very slight voltage sag before returning quickly to a sinusoidal waveform. Figure 7 plots the simulated output voltage difference between the conventional SMC and the proposed strategy under no load to full load. The conventional SMC exhibits voltage sag about nine times more than the proposed strategy, which shows its poor transient behavior. Figure 8 and Figure 9, respectively, illustrate the simulated sine wave output voltages for the conventional SMC and the proposed strategy from full load to no load. The conventional SMC lacks predictive compensation and therefore generates a tremendous voltage swell. The proposed strategy only experiences a tiny voltage swell until it returns to a perfect sine wave. The difference observed in the simulated output voltage for the conventional SMC and the proposed strategy during full load to no load are compared in Figure 10. The conventional SMC produces a voltage swell above 10% of the normal value. The proposed strategy shows quite minor voltage swell, almost equivalent to the reference level. Figure 11 and Figure 12 depict the simulated sine wave output voltage utilizing both the conventional SMC and the proposed strategy at rectified loading, respectively. The proposed strategy approximates the sinusoidal very closely, revealing a well-stable performance with a THD of 0.46%. The conventional SMC suffers from a severe oscillatory distortion whose THD reaches as high as 17.19%. The difference in simulated output voltage for the conventional SMC and the proposed strategy under rectified loading is illustrated in Figure 13. While the proposed strategy yields a high-quality pure sine wave, the conventional SMC imposes output voltage fluctuation within the entire sinusoidal curve. Figure 14 provides a comparison of simulated DC output voltage from the converter between the conventional SMC and the proposed strategy under partial shading. The output of the proposed strategy is nearly the DC link voltage value. However, the conventional SMC lacks the capability of state prediction, which causes a reduction in DC voltage during partial shading. Figure 15 displays the experimental sine wave output voltage obtained using the conventional SMC from no load to full load. Figure 16 shows the experimental sine wave output voltage of the proposed strategy subjected to similar loading situation. Contrasting with the conventional SMC, the proposed strategy produces very low voltage sag resulting in a shorter restore period. Figure 17 analyzes the experimental output voltage difference between the conventional SMC and the proposed strategy for no load to full load. The voltage sag of the conventional SMC greatly exceeds that of the proposed strategy and fails to recover towards the reference voltage. The experimental sine wave output voltages of the conventional SMC and the proposed strategy from full load to no load are demonstrated in Figure 18 and Figure 19, respectively. The conventional SMC produces about four times the voltage swell of the proposed strategy, causing an extremely weak transient behavior. The comparison of the experimental output voltage difference between the conventional SMC and the proposed strategy for full load to no load is indicated in Figure 20. The voltage swell of the proposed strategy satisfies industry standard, but the conventional SMC causes an influence on the power quality. Figure 21 reveals the experimental sine wave output voltage of the conventional SMC operating at rectified loading. The THD value goes up to 20.54%, showing a distorted non-sinusoidal waveform. The experimental sine wave output voltage with the proposed strategy for the same loading is presented in Figure 22. It produces a nearly matching waveform to the referenced sinusoidal voltage, with a THD value as low as 0.53%, delivering great steady state. A difference comparison of the experimental output voltages with the conventional SMC and the proposed strategy under rectified loading is presented in Figure 23. The proposed strategy yields a nice steady-state AC voltage, while the conventional SMC displays sine curve deformation distortion. The experimental DC output voltage of the converter for the conventional SMC and the proposed strategy are comparatively shown in Figure 24 under partial shading. The proposed converter still outputs almost the same voltage as the DC link value during partial shading. The conventional SMC suffers from a lack of control force, leading to a noticeable drop in DC output voltage. Table 2 summarizes the comparison of simulated and experimental voltage sag, voltage swell, and THD levels for the conventional SMC and the proposed strategy at various loadings. It is noteworthy that the conventional SMC uses the signum function, which has a greater force of control. The percentage difference in voltage sag and swell between simulated and experimental data may be small. However, the conventional SMC suffers from the chattering phenomenon. The chattering will trigger un-modeled high-frequency system dynamics and induce transistor heating, as well as mechanical vibration and wear. When not considered as a percentage difference, the conventional SMC still does not meet the IEEE 1159-2019 standard [35] for voltage sag and swell. In order to improve the chattering, the smoothing function and the GLRMs are contained within the proposed strategy. The control force is weakened so that a slightly larger percentage difference than the conventional SMC may occur. However, the proposed strategy exceeds the IEEE 1159-2019 standard for voltage sag and swell. From the point of view of obtaining a high-quality AC power supply, the proposed strategy is better than the conventional SMC.

## 5. Discussion

Previous works about SMC applied to inverters can be discussed. The concept of the optimization of coupling a discrete-time SMC and feed-forward solution is presented to achieve improved tracking behavior of PWM inverters. Under rectifier load cases, it is impossible to accurately arrive at the required sliding surface. The output voltage waveform distorts apparently [36]. The UPS (uninterruptible power supply) inverter with variable structure SMC is suggested. The proposed inverter employs multi-loop control as well as a smoothing function. However, the insufficient control strength causes an unsatisfactory transient response for drastic load changing [37]. A discrete integral variable structure control is proposed [38]. The waveform becomes distorted at rectified loads, which is the shortcoming caused by the remaining effect of the chattering [9]. Therefore, the simulation and experimental results of the proposed strategy surpass the above-mentioned prior works. Regardless of voltage sag or swell or THD, the output voltage of the proposed inverter is compliant to the IEEE (Institute of Electrical and Electronics Engineers) 519-2014 [39] and IEEE 1159-2019 standards.

Some full-order advanced SMC techniques have been exploited to provide smoothing of the control signal. It can limit chattering and increase the robustness of the control system, which is an interesting issue presented below. The full-order terminal sliding mode control with no chattering has been put forward for addressing the rectifier and inverter sites of the soft open point. The current loop forces the current response to track its reference value for a finite period of time. In addition, it allows the power loop as a way towards achieving precision in power following in the event of interfering circumstances [40]. A robust stepwise full-order sliding mode of voltage control structure is proposed for stand-alone single-phase inverters capable of interacting with green energy resources. The offered controller adopts a cascading approach with external, as well as internal, loops, allowing for tracking of the requested loading voltage. The full-order sliding manifolds can mitigate the chatter and contribute to the transience and steady-state response, minimizing harmonic distortion [41]. The recent publications on grey prediction enhance the forecasting quality by associating wavelet transformed signal analysis methods and artificial intelligence learning with neural networks. Such a subject is examined to be beneficial in forecasting systems containing uncertainties. There is a discrete grey multivariate convolution model with an optimum solution being developed, as well as practiced. The predictive modeling process relies on the discreet grey model together with wavelet based data filtration technology. By presenting annually the Cameroonian power requirements, the suggested model possesses both greater predictive accuracy as well as considerably more robustness [42]. The nonlinear grey wavelet support vector regression model is presented in response to the sophisticated characteristics in Chinese gas data sets. It allows fitting complicated information modes and addressing both unusual values and noise information to improve the forecasting precision. Findings illustrate that the model clearly outperforms all alternative models with respect to out-of-sample forecasting [43]. The presented model is suggested to forecast the surface remaining sedimentation of the goaf. It provides a more advanced forecasting precision over a unitary model and conforms to the variation rule of the observed values [44].

## 6. Conclusions

The specific strengths of the NISMC contribute to quick limited-time state convergence, decreased chattering, and reduced steady-state errors, thereby boosting exact tracking control. As well, the GLRM is used to find the global maximum power point. The proposed strategy can optimize the power output of photovoltaic energy conversion systems whilst maintaining the maximum energy converting efficiency under uncertain disturbances. During the circumstances of abrupt load connection, abrupt load disconnection, and rectified loading, the proposed strategy is able to deliver remarkable transience and steady state. These good tracking responses include very low voltage sag, voltage swell, and total harmonic distortion. However, the output–voltage behavior using the conventional SMC outperforms the industry standard when subjected to the same testing conditions, bringing unsatisfactory quality. Simulation and experimental results have witnessed the high performance of the proposed strategy in various types of loading.

## Figures and Tables

**Table 1:** Parameters of the pure sine wave inverter.

System Parameters	Values
DC-link voltage ($V_{d}$)	200 V
Sine wave output voltage ($v_{ac}$)	110 V_rms_
Frequency of sine wave output voltage	60 Hz
Filter inductor (L)	0.2 mH
Filter capacitor (C)	20 μF
Resistive load ($R_{L}$)	12 ohm
Switching frequency	30 kHz
Control Parameters	
$\kappa_{1}/\kappa_{2}=5/3$, $\gamma_{1}=0.3$, $\gamma_{2}=1.7$, $\gamma_{3}=0.6$, ζ=0.03, and δ=0.02.	

**Table 2:** Comparison of voltage sag, voltage swell, and THD.

Conventional SMC			
Simulations	Abrupt load increasing	Abrupt load removing	Rectified loading
	Voltage sag	Voltage swell	THD
	69.93 V	16.14 V	17.19%
Proposed strategy			
Simulations	Abrupt load increasing	Abrupt load removing	Rectified loading
	Voltage sag	Voltage swell	THD
	7.96 V	2.23 V	0.46%
Conventional SMC			
Experiments	Abrupt load increasing	Abrupt load removing	Rectified loading
	Voltage sag	Voltage swell	THD
	67.98 V	19.43 V	20.54%
Proposed strategy			
Experiments	Abrupt load increasing	Abrupt load removing	Rectified loading
	Voltage sag	Voltage swell	THD
	10.85 V	4.42 V	0.53%

## Data Availability

The data are contained within the article.
